# Isolation and epithelial co-culture of mouse renal peritubular endothelial cells

**DOI:** 10.1186/s12860-014-0040-6

**Published:** 2014-11-30

**Authors:** Ye Zhao, Hong Zhao, Yun Zhang, Tania Tsatralis, Qi Cao, Ya Wang, Yiping Wang, Yuan Min Wang, Steve I Alexander, David C Harris, Guoping Zheng

**Affiliations:** Centre for Transplant and Renal Research, Westmead Millennium Institute, The University of Sydney, Sydney, NSW Australia; Department of Biochemistry and Molecular Biology, Shanxi Medical University, Taiyuan, PR China; Experimental Centre of Science and Research, The First Clinical Hospital of Shanxi Medical University, Taiyuan, PR China; Centre for Kidney Research, Children’s Hospital at Westmead, Sydney, NSW Australia

**Keywords:** Peritubular endothelial cells, Tubular epithelial cells, CD146, Co-culture, Vascular endothelial growth factor

## Abstract

**Background:**

Endothelial-mesenchymal transition (EndoMT) has been shown to be a major source of myofibroblasts, contributing to kidney fibrosis. However, *in vitro* study of endothelial cells often relies on culture of isolated primary endothelial cells due to the unavailability of endothelial cell lines. Our recent study suggested that peritubular endothelial cells could contribute to kidney fibrosis through EndoMT. Therefore, successful isolation and culture of mouse peritubular endothelial cells could provide a new platform for studying kidney fibrosis. This study describes an immunomagnetic separation method for the isolation of mouse renal peritubular endothelial cells using anti-CD146 MicroBeads, followed by co-culture with mouse renal proximal tubular epithelial cells to maintain endothelial phenotype.

**Results:**

Flow cytometry showed that after isolation and two days of culture, about 95% of cells were positive for endothelial-specific marker CD146. The percentage of other cells, including dendritic cells (CD11c) and macrophages (F4/80), was less than 1%. Maintenance of endothelial cell phenotype required vascular endothelial growth factor (VEGF) and co-culture with mouse proximal tubular epithelial cells.

**Conclusion:**

In this study, we established a method for the isolation of mouse renal peritubular endothelial cells by using immunomagnetic separation with anti-CD146 MicroBeads, followed by co-culture with mouse renal proximal tubular epithelial cells to maintain phenotype.

**Electronic supplementary material:**

The online version of this article (doi:10.1186/s12860-014-0040-6) contains supplementary material, which is available to authorized users.

## Background

Endothelial cells have been found to be a major source of myofibroblasts via endothelial mesenchymal transition (EndoMT), causing fibrosis in kidney and other organs [1-4]. In one key study, Zeisberg and colleagues [4] showed in three mouse models including unilateral ureteral obstructive nephropathy (UUO), Alport disease and streptozotocin-induced diabetic nephropathy, that around 30% to 50% of fibroblasts formed in the kidneys co-expressed the endothelial marker CD31 and the fibroblast/myofibroblast markers fibroblast specific protein-1 (FSP-1) and/or α-smooth muscle actin (α-SMA). Endothelial lineage tracing using Tie2-Cre;R26R-stop-EYFP transgenic mice in the UUO model [4] and in streptozotocin-induced diabetic nephropathy [5] further confirmed the presence of EndoMT-derived fibroblasts. However, elucidation of molecular mechanisms of EndoMT involving glomerular or peritubular endothelial cells relies largely on *in vitro* studies using primary isolated human or mouse endothelial cells. Such studies are limited by the loss of phenotype that occurs in those primary endothelial cells in culture after a limited number of passages.

Renal endothelial cells include glomerular endothelial cells, peritubular endothelial cells and vascular endothelial cells. Although it is generally accepted that endothelial cells contribute to fibroblast formation in kidney, the contribution of different renal endothelial cells has not been defined. Previous studies examining EndoMT in renal fibrosis were mostly focused on glomerular endothelial cells, not surprisingly using the well-established method for isolation of glomerular endothelial cells [6-9]. By immunofluorescence staining of kidney sections of mice with UUO, co-localization of the mesenchymal marker α-SMA and endothelial marker CD31 or VE-cadherin was observed predominantly outside glomeruli, suggesting that the interstitial peritubular rather than glomerular endothelial cells play the major role, at least in the UUO model. To date, however, a method for isolation of peritubular endothelial cells of high purity has not been described [10]. For example, the method described by Mcginn *et al.* [8] may isolate lymphatic and vascular endothelial cells.

Primary endothelial cells are susceptible to phenotypic change in culture; a co-culture system was, therefore, developed to mimic the *in vivo* micro-environment in the kidney with its key interactions between renal tubular epithelial cells and adjacent endothelial cells. Tasnim *et al.* [10] described interactions by which human renal glomerular endothelial cells improved the stability of the human renal tubular cell phenotype while glomerular endothelial cell phenotype was also well-maintained by tubular epithelial cells. However, such a system may not be applicable to the interaction between peritubular endothelial cells and tubular epithelial cells *in vivo*.

Here we describe a method for isolation of primary mouse renal peritubular endothelial cells (MRPEC) and co-culture with mouse renal proximal tubular epithelial cells. Our results show that separation with anti-CD146 MicroBeads resulted in peritubular endothelial cells of high purity. Further, co-culture with mouse renal proximal tubular epithelial cells maintained the phenotype of isolated peritubular endothelial cells, thus providing a stable *in vitro* model for investigating the role of peritubular endothelial cells in kidney diseases.

## Methods

### Animals

Male BALB/c mice (6 week old) were purchased from Australian Research Council and experiments were performed in accordance with protocols approved by Animal Ethics Committee of Western Sydney Local Health District.

### Separation of tubular fraction from kidney cortex

Mouse kidney tubular fractions were obtained from the kidney cortex of BALB/c mice using established methods adapted from Doctor *et al.* [11]. Kidneys were perfused *in situ* via the aorta with 20 ml phosphate buffered saline (PBS; Lonza; Walkersville, MD, USA) containing 80U/ml heparin to remove blood from anesthetized mice. Kidney capsule was removed by peeling with forceps. Freshly isolated kidneys were placed in ice-cold Dulbecco’s Modified Eagle’s Medium mixed with Ham’s F12 (DMEM/F12; 1:1 ratio; Gibco Life Technologies; Grand Island, NY, USA) on a petri dish. The kidney was sliced coronally and homogenized by mincing into 1 mm^3^ to 2 mm^3^ pieces. The homogenized kidney cortex tissue pieces were resuspended and mixed in 7.5 ml of collagenase type IV solution (Table 1) and incubated at 37°C in a gentle shaking water bath for 15 min. The suspension was homogenized by pipetting 5 to 10 times through a sterile transfer pipette followed by addition of 1 ml of fresh collagenase type IV solution. This process was repeated 2-4 times. About 40 ml fresh ice-cold DMEM/F12 was then added into the collagenase digestion solution and the suspension was centrifuged at 200 × g for 2 min. The pellet was resuspended and washed in 10 ml of fresh ice-cold DMEM/F12 and centrifuged at 150 × g for 2 min at 4°C. Density-gradient centrifugation of the pellet was then performed by resuspension in 25 ml of 45% (vol/vol) sterile Percoll solution (Table 2) in 50 ml centrifugation tubes and centrifugation at 5525 × g for 30 min at 4°C (without braking). After centrifugation, the tubule fractions were collected from the top layer of the Percoll solution (5 ml of the top layer). The tubule fraction was washed once in 20 ml ice-cold DMEM/F12 medium at 300 × g for 5 min at 4°C and resuspended for further experiments.

**Table 1:** **Components of collagenase type IV solution**

**Collagenase D solution**	**Volume or concentration**	**Supplier**
DMEM/F12 medium	7.5 ml	Gibco Life Technologies; Grand Island, NY, USA
Collagenase type IV	1 mg/ml	Sigma Chemical Co.; St Louis, MO, USA
Deoxyribonuclease	0.1 mg/ml	Sigma Chemical Co.
Bovine serum albumin (BSA)	1 mg/ml	Sigma Chemical Co.

**Table 2:** **Percoll solution (25 ml)**

**Percoll solution**	**Volume**	**Supplier**
DMEM/F12 medium	12.5 ml	Gibco Life Technologies; Grand Island, NY, USA
Percoll (45%, vol./vol.)	11.25 ml	Gibco Life Technologies
20 X concentrated PBS	0.5625 ml	AMRESCO; Solon, OH, USA
MilliQ water	0.5625 ml	Roche Applied Science; Penzberg, Upper Bavaria, Germany
1 M HEPES	80.5 μl	Gibco Life Technologies

### Isolation of proximal tubular epithelial cells from tubule fraction

The pellet was resuspended in K1 medium [DMEM:HAM’s F12; 1:1 vol/vol; Gibco Life Technologies) supplemented with 25 μg/ml of epithelial growth factor (EGF; Sigma Chemical Co.; St. Louis, MO, USA), 25 μM HEPES (Invitrogen; Carlsbad, CA, USA), hormone mixture (Table 3) and 5% fetal calf serum (FCS; Invitrogen)]. Proximal tubular epithelial cells were obtained by direct culture after removing peritubular endothelial cells.

**Table 3:** **Components of hormone mixture in K1 medium**

**Ingredient**	**Supplier**	**Stock solution**	**Vol./wt. of addition**
Insulin	Sigma Chemical Co., St. Louis, MO, USA	Powder	50 mg dissolved in 10 ml of HBSS/HEPES* and small amount of NaOH
Prostaglandin E_1_	Cayman Chemical, Ann Arbor, MI, USA	0.5 mg/ml (in EtOH)	25 μl
3,3,5-triiodothyro–nine	Sigma Chemical Co.	16.9 mg/ml (0.026 M) (in EtOH)	2 μl of stock added to 10 ml HBSS/HEPES* then used in 100 μl aliquots
Transferrin	Sigma Chemical Co.	Powder	50 mg
Sodium selenite	Sigma Chemical Co.	0.173 mg/ml (10^-6^ M) in HBSS/HEPES	100 μl
Hydrocortisone	Sigma	0.18 mg/ml (in EtOH)	1 ml

The hormone mixture was brought to a final volume of 100 ml with HBSS/HEPES, and aliquoted into 5 ml or 10 ml portions and stored at –80°C.

*HBSS/HEPES = Hanks Balanced Salt Solution without calcium and without magnesium (Gibco, Life Technologies; Grand Island, NY, USA)/1% HEPES.

### Isolation of peritubular endothelial cells from tubule fraction

The tubule fractions were further digested with 0.025% trypsin or collagenase type IV solution for 10 to 15 min at 37°C to obtain single cell suspensions. After digestion, ice-cold PBS was added to the cell suspension followed by filtering sequentially through a 70-μm and a 40-μm cell strainer. The filtered cell suspensions were then centrifuged at 300 × g for 2 min at 4°C. The pellet was resuspended in MicroBeads resuspension buffer [prepared in PBS, containing 0.5% BSA (Sigma Chemical Co.) and 2 mM EDTA (Life Technologies)] and centrifuged again at 300 × g for 3 min at 4°C. The cell pellets obtained from 2 kidneys were resuspended in 1 ml MicroBeads resuspension buffer and incubated with anti-mouse CD16/32 antibody (Fc-blocking) for 15 min to block non-specific binding. The cell pellets were then magnetically labeled with CD146 (LSEC) MicroBeads (MACS; Miltenyi Biotechnology; Bergisch Gladbach, Germany) and separated according to the manufacturer’s instructions. Specifically, cells were incubated with CD146 MicroBeads and then passed through a magnetic field. Peritubular cells were collected and cultured in the pre-warmed endothelial cell medium (ScienCell; Carlsbad, CA, USA) in fibronectin (Sigma Aldrich) pre-coated Transwell plates. Purity of the isolated peritubular endothelial cells was determined by immunofluorescent staining and flow cytometric analysis. Before FACS staining, the beads were detached from the cells using the MultiSort Release Reagent (MACS), which enzymatically removes the MicroBeads [Additional file 1].

### Flow cytometry analysis (FACS)

Adherent endothelial cells were detached from culture flasks with trypsin/EDTA (Life Technologies), washed and resuspended in PBS (Gibco Life Technologies) containing 1% FCS. Cells (10^6^ cells/ml) were then incubated with respective FITC-, PE- or APC-conjugated monoclonal antibodies for 20 min at 4°C. Fluorescence antibody-labeled cells were then washed twice in cold PBS with 1% FCS and analyzed using a flow cytometer (BD Bioscience; San Jose, CA, USA).

### Antibodies

Antibodies for flow cytometry were as follows: anti-mouse F4/80 antigen Alexa Fluor® 488, anti-mouse Lyve-1 Alexa Fluor® 488, anti-mouse CD31 (PECAM-1) APC, anti-mouse CD45 PE, anti-mouse CD11c Alexa Fluor® 488 (eBioscience; San Diego, CA, USA), PE-anti-mouse CD146, Alexa Fluor® 488 anti-mouse panendothelial cell antigen (Biolegend; San Diego, CA, USA), FITC-anti-mouse FSP-1/S100A4 (LifeSpan BioSciences; Seattle, WA, USA) and anti-mouse PDGFR-β Alexa Fluor® 488 (BD Bioscience). For isotype antibody controls, rat IgG2a κ Alexa Fluor® 488 (eBioscience) was used for F4/80, rat IgG1 κ Alexa Fluor® 488 (eBioscience) was used for Lyve-1, FSP-1 and PDGFR-β, rat IgG2a κ APC (eBioscience) was used for CD31, rat IgG2a κ PE (eBioscience) was used for CD45, Armenian hamster IgG Alexa Fluor® 488 (eBioscience) was used for CD11c, and PE rat IgG2a, κ (Biolegend) was used for CD146, and Alexa Fluor® 488 rat IgG2a, κ (Biolegend) was used for PV-1.

Primary antibodies for immunofluorescence were as follows: rabbit polyclonal anti-VE-cadherin (1:200; Alexis Biochemicals; Farmingdale, NY, USA), rabbit polyclonal anti-FSP-1/S100A4 (1:400; Merck Millipore; Billerica, MA, USA), rat monoclonal anti-CD11c (1:200; eBioscience), rat monoclonal anti-F4/80 (1:200; eBioscience), purified rat anti-CD73 (1:50; BD Bioscience), rabbit monoclonal anti-PDGFR-β (Abcam; Cambridge, UK), and mouse monoclonal anti-actin, α-smooth muscle (Sigma Chemical Co.). For isotype antibody controls, rat IgG2a κ Purified (eBioscience) was used for VE-cadherin and F4/80, rabbit IgG (Invitrogen) was used for FSP-1 and PDGFR-β, Armenian hamster IgG purified (eBioscience) was used for CD11c, Purified Mouse IgG1, κ (BD Bioscience) was used for CD73, and mouse IgG2a, κ (Biolegend) was used for α-SMA.

Secondary antibodies for immunofluorescence were as follows: fluorescent-conjugated secondary anti-rabbit Alexa Fluor® 488 (1:600; Invitrogen), anti-mouse Alexa Fluor® 546 (1:600; Invitrogen) and anti-rat Alexa Fluor® 488 (1:600; Invitrogen).

### Cell culture

The primary mouse peritubular endothelial cells isolated using MicroBeads were cultured in endothelial cell medium containing VEGF (Sigma-Aldrich; St. Louis, MO, USA) (2.5 μg/ml to 5 μg/ml) according to a previously described method [6]. The endothelial cell medium contained 93% of basal medium (ScienCell), 5% fetal bovine serum (FBS; ScienCell), 1% endothelial cell growth supplement (ECGS; ScienCell) and 1% penicillin/streptomycin (P/S; ScienCell). Cells were maintained at 37°C in 5% CO_2_ and incubated overnight in plates pre-coated with fibronectin in endothelial cell medium, non-adherent cells were removed and medium was changed. Then medium was changed every 2-3 days. Experiments with mono-cultures and co-cultures were performed in fibronectin precoated 6-well Transwell plates (BD Bioscience) with polyester inserts (pore size 0.4 μm) with endothelial cell medium. The proximal tubular epithelial cells from tubule fractions were incubated overnight in plates in K1 medium at 37°C with 5% CO_2_, non-adherent cells were removed and medium was changed. Then medium was changed every 2-3 days. In the co-culture system, peritubular endothelial cells were placed in the bottom chamber of Transwell plates while proximal tubular epithelial cells were placed in polyester inserts and cultured in endothelial cell medium.

### Immunofluorescence

For immunofluorescent staining, cells were seeded on glass coverslips in 6-well culture plates and cultured until they reached 50% to 60% confluence. Cells were then washed with PBS, fixed with absolute methanol for 10 min at -20°C, and blocked with 2% BSA (Sigma Chemical Co.) for 1 h at room temperature. The coverslips were incubated for 1 h at room temperature with primary antibodies against endothelial cell marker VE-cadherin, fibroblast cell marker FSP-1, dendritic cell marker CD11c, macrophage cell marker F4/80, perivascular fibroblast marker CD73, or pericyte specific marker PDGFR-β. After washing in PBS and distilled water, cells were incubated with fluorescent-conjugated secondary antibody for 40 min at room temperature in the dark. The coverslips were then washed in PBS and distilled water and counterstained with 4′,6-diamidino-2-phenylindole (DAPI) (Invitrogen) for 5 min. After washing in PBS and distilled water, the coverslips were mounted with fluorescence mounting medium (Dako; Glostrup, Denmark) and subjected to fluorescence microscopy. Isotype controls of corresponding secondary antibodies were used as negative controls.

### Semiquantitative assessment of immunofluorescent staining

A minimum of 10 consecutive fields of immunofluorescent images were taken from each slide of stained MRPECs with total cell count of at least 2000 cells. Results were obtained from duplicate slides of a minimum three independent experiments. The number of positive staining cells was counted according to the number of nuclei counterstained by DAPI (blue).

### Statistical analysis

Results from at least three independent experiments were expressed as mean ± SEM. Statistical significance was evaluated using a two-tail t-test for comparison between two groups. A value of *P* < 0.05 was considered statistically significant.

## Results

### Anti-CD146 MicroBeads isolation of peritubular endothelial cells from the tubule fraction of kidney cortex

**Figure 1:**
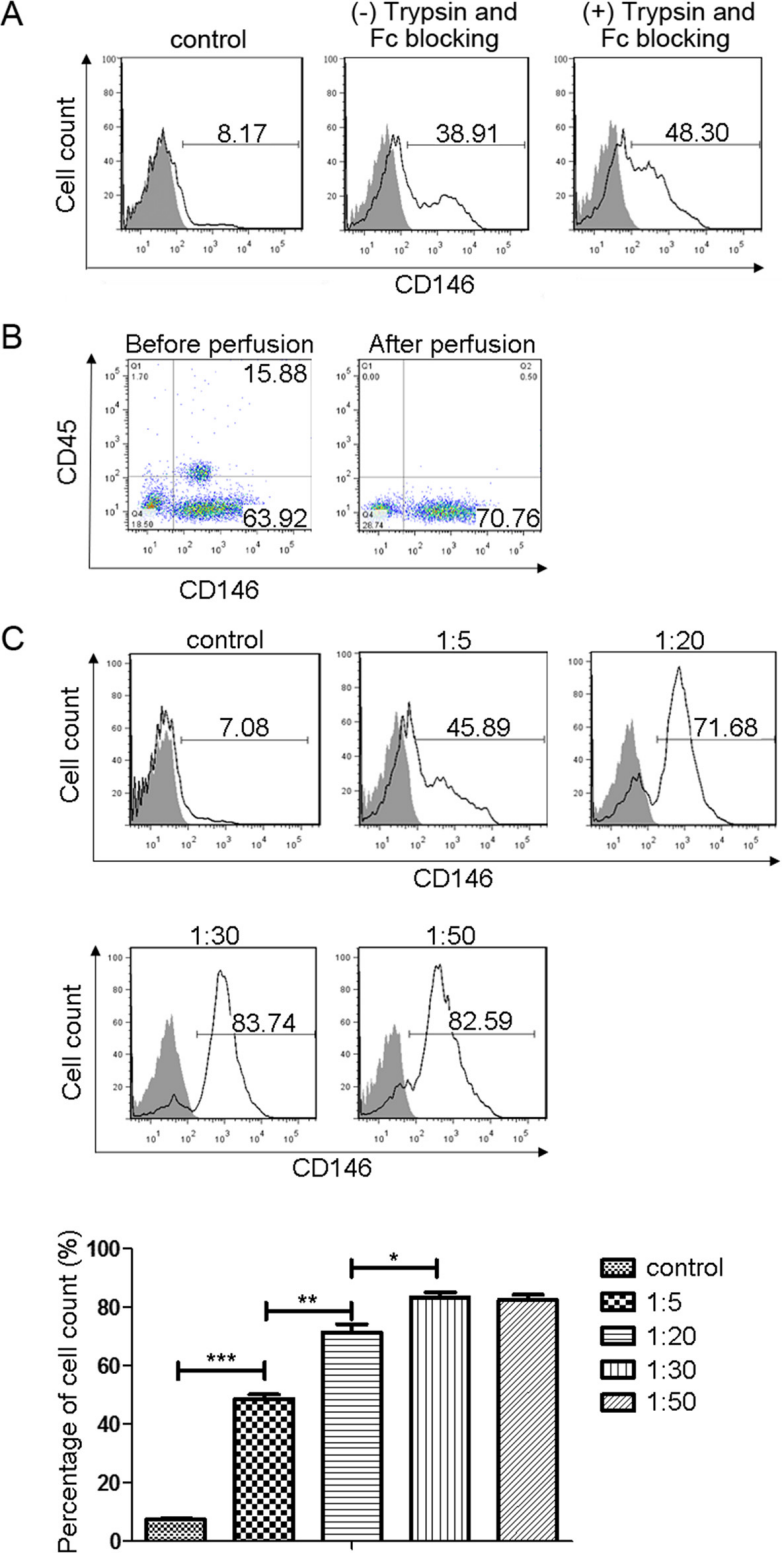
**Flow cytometric analysis of peritubular endothelial cells isolated by MicroBeads. (A)** Flow cytometry analysis of CD146-PE positive endothelial cells before (left panel), after MicroBeads isolation (middle panel), and after MicroBeads isolation with Fc-blocking and trypsin digestion (right panel). Shaded areas are respective isotype controls. **(B)** Flow cytometry analysis of blood cell (CD45) contamination when CD146-PE positive endothelial cells were isolated with MicroBeads (1:20) with (right panel) or without kidney perfusion (left panel). Shaded areas are respective isotype controls. **(C)** After perfusion, CD146-PE positive cells before (top left panel) and after MicroBeads isolation at different MicroBeads concentration: 1:5 (top middle panel), 1:20 (top right panel), 1:30 (bottom left panel) and 1:50 (bottom right panel). Shaded areas are respective isotype controls. (G) Statistical analysis of each individual experiment (N = 3). After modification of kidney perfusion and optimization of MicroBeads concentration, the purity of CD146 positive cells increased significantly from 7.5 ± 0.3 (before separation) to as high as 83.3 ± 1.7. Data are expressed as mean ± SEM with N = 3 for each experimental group. *P < 0.05, **P < 0.01, ***P < 0.001.

Following isolation using anti-CD146 MicroBeads, the peritubular endothelial cells were assessed by FACS analysis. The FACS results show that before magnetic separation, the percentage of CD146-positive cells was around 8% (Figure 1A, left panel). The percentage of CD146-positive cells was around 38% after magnetic separation (Figure 1A, middle panel). To improve purity, we used Fc-blocking and typsinisation. Fc-blocking was used in our isolation of peritubular endothelial cells with anti-CD146 (rat anti-mouse) MicroBeads as described [12] to avoid non-specific binding. To obtain single-cell suspensions from MicroBeads isolation, the tubule fraction underwent digestion with optimized concentrations of trypsin to avoid co-sorting of non-endothelial cells by sorting of endothelial-non-endothelial cell aggregates. The FACS results showed that Fc-blocking and trypsin digestion increased the percentage of CD146 positive endothelial cells from 39% (Figure 1A, middle panel) to 45% (Figure 1A, right panel).

### MicroBeads concentration optimization and kidney perfusion to increase purity of isolated peritubular endothelial cells

After MicroBeads (1:20) isolation, 15% of cells were CD45+ (Figure 1B, middle panel), suggesting contamination with blood cells. Therefore, we performed kidney perfusion before digestion to remove blood cell. The peritubular endothelial cell purity thus was increased to 70% and CD45 positive cells reduced to around 1% (Figure 1B, right panel). To further improve the purity, the MicroBeads to cell suspension ratio was optimized. After kidney perfusion, when MicroBeads to cell suspension ratio was changed from 1:5 to 1:30 (Figure 1C, top panels), the purity of CD146 positive endothelial cells was increased to >80% (Figure 1C, bottom left panel) while the purity by further dilution at a ratio of 1:50 remained at the same level (Figure 1C, bottom right panel). So the final ratio of MicroBeads to cell suspension used was 1:30.

### 2 days of culture after MicroBeads isolation to increase purity of peritubular endothelial cell

**Figure 2:**
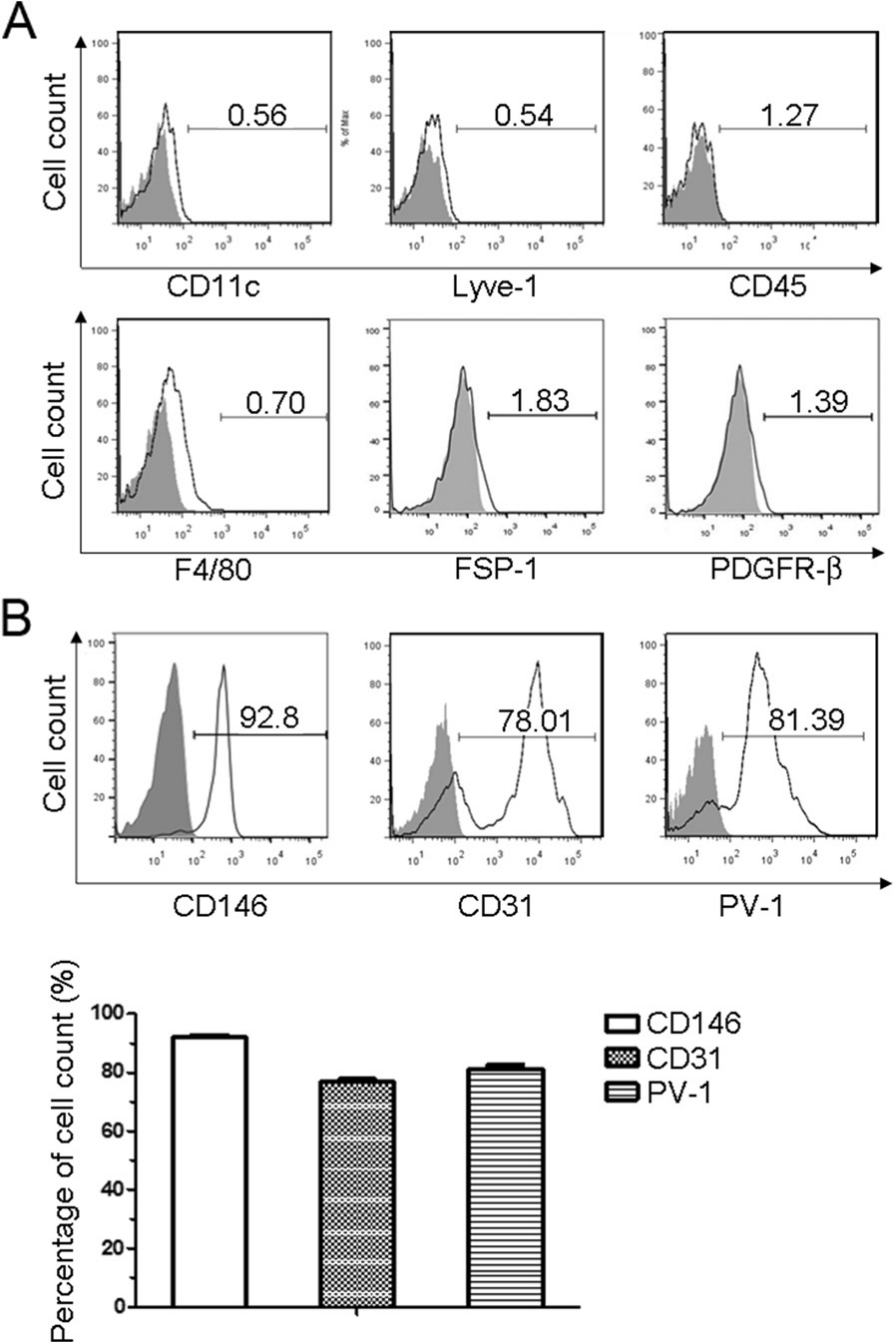
**Assessments of purity of cultured endothelial cells. (A)** Flow cytometry analysis of CD11c, Lyve-1, CD45, F4/80, FSP-1 and PDGFR-β positive cells in MicroBeads isolated peritubular endothelial cells after 2 days of culture. Shaded areas are respective isotype controls. **(B)** Flow cytometry analysis of the MicroBeads isolated peritubular endothelial cells by CD146, CD31 and PV-1 after 2 days of culture with statistical analysis (N = 3 independent experiments) (bottom panel).

After incubation with the optimal concentration of MicroBeads (1:30) and following immunomagnetic separation, the percentage of CD146-positive cells remained stable at about 80%. Magnetically isolated peritubular endothelial cells were cultured for two days and then analyzed with antibodies for different cell surface markers to determine the purity of endothelial cells and possible contamination of other cells by FACS. The peritubular endothelial cells were incubated with antibodies for lymphatic vessel endothelial cell marker Lyve-1, dendritic cell marker CD11c, hematopoietic cell marker CD45, macrophage cell marker F4/80, fibroblast cell marker FSP-1, pericyte cell marker PDFGR-β, and endothelial cell markers CD146 and CD31. To distinguish from glomerular endothelial cells, PV-1 was used as peritubular endothelial marker [13]. The FACS results showed that the respective percentage for other cell type was less than 1.83% (Figure 2A). The percentage of CD31 positive cells was 78% and that of CD146 positive cells was 92% (Figure 2B). These results could be due to CD31 being trypsin sensitive [12] while CD146 is trypsin resistant. However, the percentage of contaminating cells could potentially be higher if their surface markers were trypsin sensitive. The results after 2 days culture suggested that culturing of isolated peritubular endothelial cells further improved their purity, possibly through separation of adherent endothelial cells from other non-adherent cells. Over 2 million cells were obtained from each mouse.

### Immunofluorescence staining to show high purity of isolated mouse renal peritubular endothelial cells (MRPECs)

**Figure 3:**
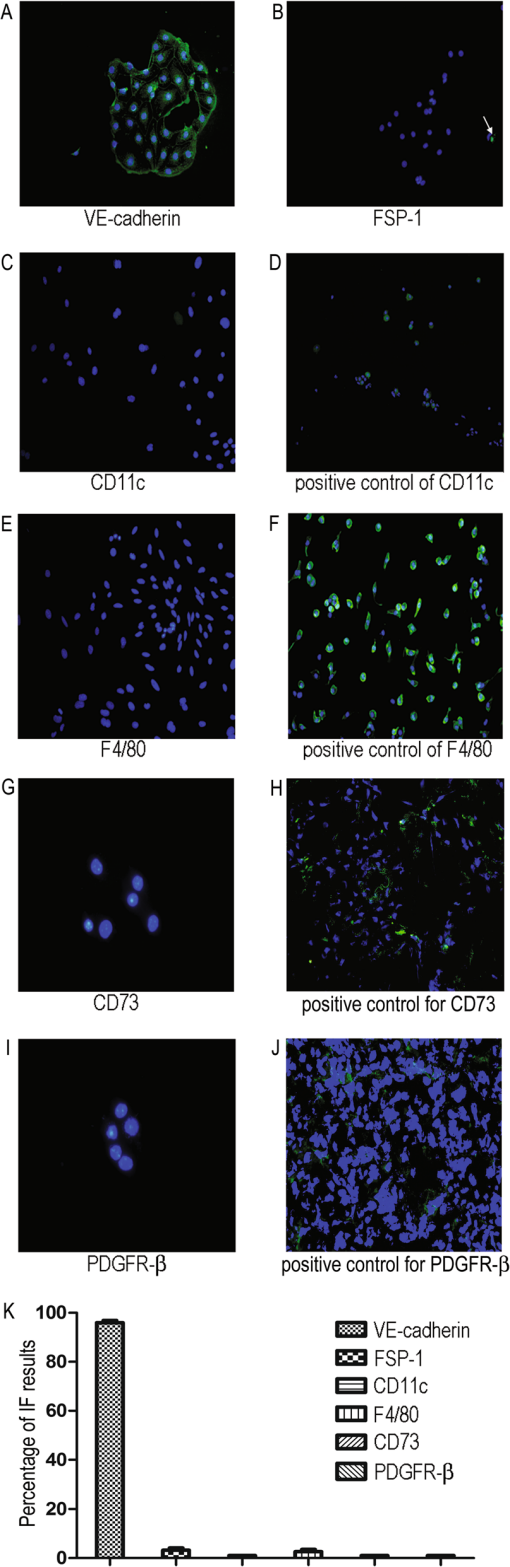
**Immunofluorescence staining assessment of MicroBeads isolated peritubular endothelial cells after 2 days culture.** Immunofluorescence staining of isolated peritubular endothelial cells against VE-cadherin (green, **A**), FSP-1 (green, indicated by white arrow, **B**), CD11c (green, **C**), F4/80 (green, **E**), and positive control for CD11c (primary spleen dendritic cells, green, **D**) and F4/80 (primary spleen macrophage, green, **F**), Immunofluorescence staining against pericyte marker CD73 (green, **G**) and PDGFR-β (green, **I**), and positive control for CD73 (frozen kidney sections of BALB/c mouse, green, **H**) and PDGFR-β (frozen kidney sections of BALB/c mouse, green, **J**). **(K)** Statistical analysis of each individual marker immunofluorescent stained and quantified as a percentage of positively stained cells against total cells (2000). Data are expressed as mean ± SEM with N = 10 for each experimental group. Original magnification was x 200; original magnification for pericytes staining was x 400. Cells in this figure were counterstained with DAPI to visualize nuclei (blue).

The high purity of isolated MRPECs shown by FACS analysis was further confirmed with immunofluorescent staining. After two days of culture, immunofluorescent staining of isolated MRPECs showed that all cells were VE-cadherin positive and few cells were FSP-1 positive (Figure 3A, B). Immunofluorescent staining for CD11c and F4/80 also suggested that no dendritic or macrophage cells were present (Figure 3C, E). To confirm the absence of pericyte contamination, cells were stained for CD73 and PDGFR-β. Results showed that almost none of the cells expressed CD73 or PDGFR-β (Figure 3G, I). Therefore, taken together the FACS analysis and immunofluorescent results showed that MRPECs of high purity were successfully isolated from mouse renal tissue.

### Co-culture with proximal tubular epithelial cells and VEGF to prevent phenotypic change of isolated peritubular endothelial cells

**Figure 4:**
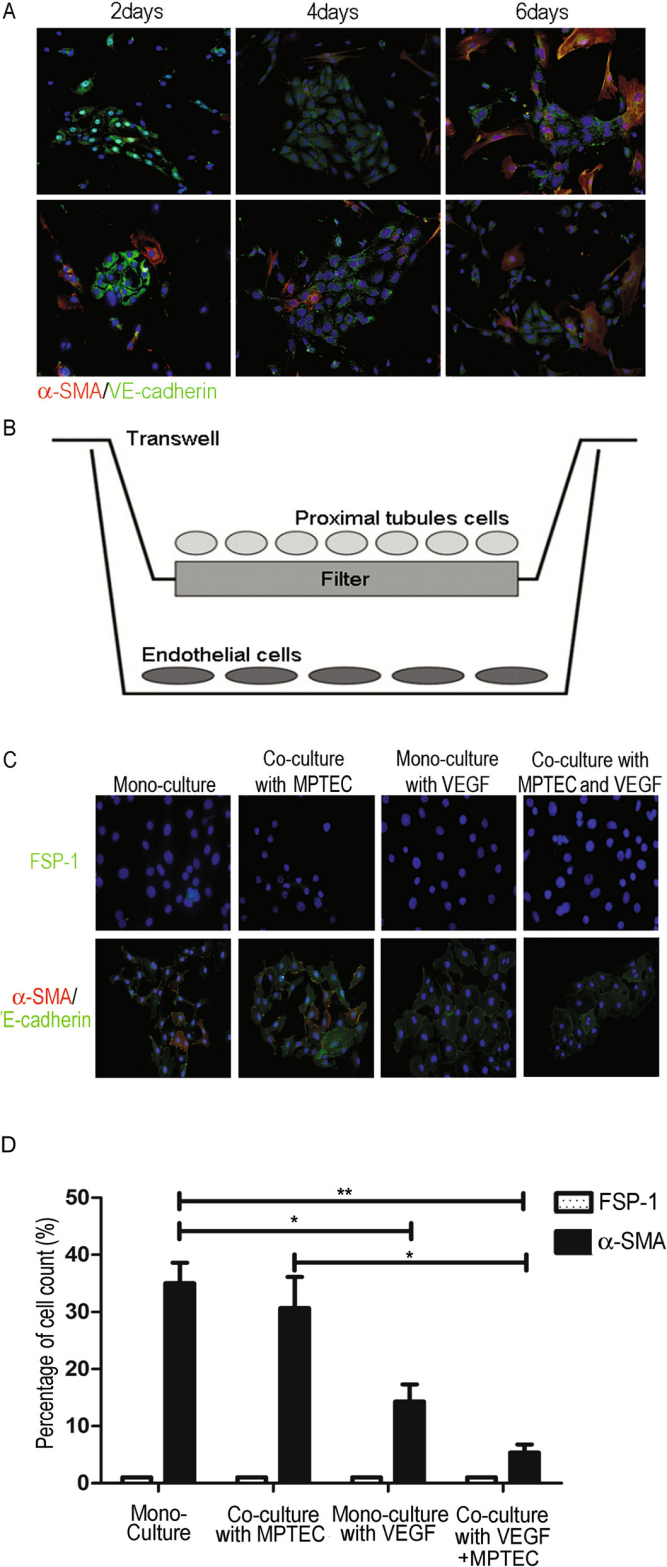
**Primary mouse renal peritubular endothelial cell performance in mono- and co-culture with mouse proximal tubular epithelial cell over 6 days. (A)** Co-localization of VE-cadherin (green) and α-SMA (red) positive cells cultured in MPRECs mono-cultures without VEGF. **(B)** Peritubular endothelial cell and proximal tubular epithelial cell co-culture. In the co-culture system, isolated peritubular endothelial cells were placed in the bottom chamber of fibronectin pre-coated Transwell plates and proximal tubular epithelial cells were seeded onto the polyester inserts (pore size 0.4 μm). Endothelial cell medium was used for the co-culture. **(C)** After 6 days, primary mouse renal peritubular endothelial cell performance in mono- and co-culture in the presence or absence of VEGF was assessed by FSP-1(green, top panels) staining and co-localization of α-SMA (red) and VE-cadherin (green, bottom panels). Orange represents α-SMA and VE-cadherin double positive areas. **(D)** Statistical analysis of primary mouse renal peritubular endothelial cell performance after cells were cultured for 6 days. FSP-1 (green) and α-SMA (red) expression in MRPECs was quantified and expressed as a percentage of positive stained cells against total cells. Images presented are representative of at least 5 independent replicate experiments. Data are expressed as mean ± SEM with N = 5 for each group. *P < 0.05, **P < 0.01 vs. respective control. Original magnification was x 200. Cells in this figure were counterstained with DAPI to visualize nuclei (blue).

Immunofluorescence staining of MRPECs cultured for 2, 4 and 6 days showed VE-cadherin positive staining. However, a large number of cells were also α-SMA positive after 2 days of culture, suggesting that the cells were undergoing phenotypic change (Figure 4A). To maintain MRPEC endothelial phenotype, vascular endothelial growth factor (VEGF) was added to the culture medium and MRPECs were co-cultured with mouse proximal tubular epithelial cells (MPTECs) which were obtained via direct culture of tubule fractions (Figure 4B). FSP-1 and α-SMA were used as markers for fibroblasts [4]. While FSP-1 (Figure 4C, top panels) staining was low in all groups, α-SMA staining co-localized with VE-cadherin (Figure 4C, bottom panels) significantly diminished in culture of peritubular endothelial cells after treatment with VEGF and especially when combined in co-culture with proximal tubular epithelial cells (Figure 4C, D). Statistical analysis showed the effect of co-culture of MRPECs with MPTECs was minimal as compared to mono-culture of MRPECs without any treatment (Figure 4D). However, α-SMA expression decreased significantly when the cells were treated with VEGF (from 35.0 ± 3.6 to 14.3 ± 3.0, *P* <0.05). When the cells were co-cultured with MPTECs and treated with VEGF, the number of α-SMA positive cells was the lowest among all groups (from 35.0 ± 3.6 to 5.3 ± 1.5, *P* < 0.001) (Figure 4D). This result demonstrated that co-culture with MPTECs and VEGF treatment is an effective method for maintaining isolated MRPECs.

## Discussion

Anti-CD146-conjugated MicroBeads have been used for isolation of endothelial cells from liver [14]. Anti-CD31 was previously used in isolation of endothelial cells. However, pericytes are also positive for CD31. In addition, endothelial surface expression of CD31 could be decreased by trypsin digestion during isolation as demonstrated in our current study. Therefore, we used for the first time anti-CD146 MicroBeads to isolate peritubular endothelial cells. Our results showed that the percentage of CD146-positive cells was as high as 92% while that of CD31-positive cells was up to 80%. To separate peritubular endothelial cells from pericytes, which may also be positive for CD146, we isolated peritubular cells using an established method for high-purity tubules isolation [15] to avoid pericyte contamination. Isolated peritubular endothelial cells stained negatively for the pericyte markers CD73 and PDGFR-β [16], while they were positive for VE-cadherin.

To improve the purity of cells isolated from tubular fractions with trypsin-EDTA digestion, treatment of the suspensions with Fc-blocking reagents (mouse Ig) is needed to avoid contamination with the other types of cells that express Fc receptor. Marelli-Berg *et al.* [12] showed that treatment with Fc-blocking reagents is essential for high specificity and efficiency during positive selection, particularly when using antibodies derived from a closely related species (e.g. rat anti-mouse).

Enzymatic digestion, particularly with trypsin-EDTA, has been reported by Marelli-Berg and colleagues to be capable of lowering surface level of CD31 [12]. They showed that much higher levels of CD31 could be detected on endothelial cells as compared to those endothelial cells after treatment with trypsin–EDTA solution to detach them from the culture flasks. This may account for the difference between CD31 and CD146 staining (80% vs. 92%) for isolated peritubular endothelial cells. However, trypsin treatment is necessary for preparation of single cell suspensions. In addition, we optimized the trypsin concentration to minimize the influence on other cell surface markers.

Renal peritubular endothelial cells are, however, susceptible to undergoing phenotypic change in culture. Manual removal of phenotype-changed cells [8] is technically difficult to optimize. A co-culture system was, therefore, developed to mimic the *in vivo* micro-environment of the kidney in which the interactions between renal tubular epithelial cells and adjacent endothelial cells are vital for survival of peritubular endothelial cells [10]. VEGF was used previously to aid maintenance of endothelial cell phenotype [6]. Our results proved that VEGF can help MRPECs maintain their phenotype, possibly because VEGF has profound effects on endothelial cells and stimulates their survival and proliferation, as well as vasculogenesis and angiogenesis [17,18]. Furthermore, co-culture with proximal tubular epithelial cells increased the stability of the endothelial phenotype beyond that achieved by incubation with VEGF alone. It is possible that renal epithelial cells and endothelial cells formed a micro-environment that positively affects both cell types and promotes survival and proliferation.

The potentially critical interactions between epithelial and endothelial cells and soluble factors in the micro-environment have been described in a study by Tasnim and Zink [10]. They showed that co-culture with proximal tubular epithelial cells stimulated glomerular endothelial cells to express increased amounts of hepatocyte growth factor (HGF) and VEGF. In addition, endothelial cells also secreted increased amounts of TGF-β1 and its antagonist α2-macroglobulin (A2M) in the presence of proximal tubular epithelial cells. A2M balanced the effects of TGF-β1 and the long-term maintenance of renal epithelia was improved in the presence of HGF and VEGF [19,20]. Here we described a co-culture of tubular epithelial cells with peritubular endothelial cells. Although the mechanism remains to be explored, peritubular rather than glomerular endothelial cells are the endothelial cells that interact with tubular epithelial cells *in vivo* in kidney. Thus, the co-culture system better reflects the physiological environment of peritubular endothelial cells.

## Conclusions

We have successfully isolated peritubular endothelial cells from mouse renal tissue with high purity and maintained their phenotype in a co-culture system. The methods of peritubular endothelial cell isolation and co-culture established in this study should provide an *in vitro* model for investigation the role of peritubular endothelial cells in kidney fibrosis and all other tubulointerstitial kidney diseases.

## Additional file

Additional file 1:
**Procedures for isolation of MRPECs.**

## Abbreviations

A2M: α2-macroglobulin
CKD: Chronic kidney disease
DAPI: 4′,6-diamidino-2-phenylindole
ECGS: Endothelial cell growth supplement
EndoMT: Endothelial mesenchymal transition
HGF: Hepatocyte growth factor
FBS: Fetal bovine serum
FCS: Fetal calf serum
FSP-1: Fibroblast specific protein-1
HGF: Hepatocyte growth factor
MRPEC: Mouse renal peritubular endothelial cell
MPTEC: Mouse proximal tubular epithelial cell
PBS: Phosphate buffered saline
P/S: Penicillin/streptomycin
α-SMA: α-smooth muscle actin
UUO: Unilateral ureteral obstruction
VE-cadherin: Vascular endothelial cadherin
VEGF: Vascular endothelial growth factor
