# Supplementary material for: Isolation and epithelial co-culture of mouse renal peritubular endothelial cells
Source: BMC Cell Biol. 2014 Nov 30;15:40. doi: 10.1186/s12860-014-0040-6 (PMC4260259; doi:10.1186/s12860-014-0040-6)
Supplement: Additional file 1: — Procedures for isolation of MRPECs. [file 12860_2014_40_MOESM1_ESM.docx]

**Supplementary methods**

1. Kidneys were perfused *in situ* via the aorta with 20 ml phosphate buffered saline containing 80 U/ml heparin to remove blood from anesthetized mice.
2. Kidney capsule was removed by peeling with forceps.
3. Freshly isolated kidneys were placed in ice-cold Dulbecco’s Modified Eagle’s Medium mixed with Ham’s F12 on a petri dish.
4. The kidney was sliced coronally and homogenized by mincing into 1 mm^3^ to 2 mm^3^ pieces.
5. The homogenized kidney cortex from each mouse was resuspended and mixed in 7.5 ml of collagenase type IV solution.
6. Minced kidney was incubated at 37˚C in a gentle shaking water bath for 15 min.
7. The suspension was homogenized by pipetting 5 to 10 times through a sterile transfer pipette followed by addition of 1 ml of fresh collagenase type IV solution (repeated 2-4 times).
8. About 40 ml fresh ice-cold DMEM/F12 was then added into the collagenase digestion solution and the suspension was centrifuged at 200 x g for 2 min.
9. The pellet was resuspended and washed in 10 ml of fresh ice-cold DMEM/F12 and centrifuged at 150 x g for 2 min at 4˚C.
10. Density-gradient centrifugation of the pellet was then performed by resuspension in 25 ml of 45% (vol/vol) sterile Percoll solution in 50 ml centrifugation tubes and centrifugation at 5525 x g for 30 min at 4˚C (without braking).
11. After centrifugation, the tubule fractions were then collected from the top layer of the Percoll solution (5 ml of the top most layer).
12. The tubule fraction was washed once in 20 ml ice-cold DMEM/F12 medium at 300 x g for 5 min at 4˚C.
13. **Isolation of proximal tubular epithelial cells from tubule fraction**

Pellet was resuspended in K1 medium for proximal tubular epithelial cells culture (For further use in co-culture).

1. **Isolation of peritubular endothelial cells from tubule fraction**

The tubule fractions were further digested with 0.025% trypsin for 10 min at 37˚C.

1. After digestion, ice-cold PBS was added to the cell suspension followed by filtering sequentially through a 70-μm and a 40-μm cell strainer.
2. The filtered cell suspensions were then centrifuged at 300 x g for 2 min at 4˚C.
3. The pellet was resuspended in MicroBeads resuspension buffer and centrifuged again at 300 x g for 3 min at 4˚C.
4. The cell pellets were resuspended in 1 ml MicroBeads resuspension buffer and incubated with anti-mouse CD16/32 antibody (Fc-blocking) for 15 min to block non-specific binding.
5. The cell pellets were then magnetically labeled with CD146 (LSEC) MicroBeads and separated according to the manufacturer’s instructions. Specifically, cells were incubated with CD146 MicroBeads and then passed through a magnetic field.
6. Peritubular cells were collected and cultured in the pre-warmed endothelial cell medium in fibronectin pre-coated Transwell.
